# Effectiveness of Dietary Intervention with Iron and Vitamin C Administered Separately in Improving Iron Status in Young Women

**DOI:** 10.3390/ijerph191911877

**Published:** 2022-09-20

**Authors:** Dominika Skolmowska, Dominika Głąbska

**Affiliations:** Department of Dietetics, Institute of Human Nutrition Sciences, Warsaw University of Life Sciences (SGGW-WULS), 159C Nowoursynowska Street, 02-776 Warsaw, Poland

**Keywords:** dietary intervention, plant-based dietary intervention, non-heme iron, iron-fortified products, vitamin C, iron status, iron absorption, young women

## Abstract

In order to maintain an adequate iron status in young women, effective dietary interventions should provide sufficient amounts of iron in everyday meals and improve the bioavailability of non-heme iron by providing vitamin C. While some intervention studies administering products rich in vitamin C in conjunction with iron sources showed improved iron status, it is unknown whether a separate administration of products rich in iron and vitamin C may be a successful strategy as well. The aim of this study was to assess the effectiveness of dietary intervention with iron and vitamin C administered separately in improving iron status in young women to prevent iron deficiency anemia. The study was conducted in a group of 29 women aged 18–30, and an 8-week dietary intervention was performed. Study participants with an adequate iron status received 50 g of iron-fortified oat flakes (as a source of non-heme iron) with breakfast and 200 mL of orange juice (as a source of vitamin C) in the second part of the day. Iron status was analyzed based on red blood cells, hemoglobin, hematocrit, serum ferritin, and serum iron, and it was assessed at baseline, after 4 weeks, and after 8 weeks of the intervention. The intakes of iron, vitamin C, and folate were controlled throughout the study period, and menstrual blood loss was estimated. After 8 weeks of intervention, statistically significant differences compared with baseline were observed only for hematocrit, as its level after 8 weeks of intervention was higher than the baseline (*p* = 0.0491). Comparing subsamples within the dietary intervention considered effective and ineffective for red blood cell levels, it was indicated that lower baseline vitamin C intake may result in a more effective dietary intervention (*p* = 0.0231). Comparing subsamples within the dietary intervention considered effective and ineffective for hemoglobin, hematocrit, iron, and serum ferritin levels, it was indicated that higher baseline levels of hemoglobin (*p* = 0.0143), hematocrit (*p* = 0.0497), iron (*p* = 0.0101), and serum ferritin (*p* = 0.0343) respectively may result in a more effective dietary intervention. It was concluded that dietary intervention with iron and vitamin C administered separately may be effective in improving iron status in young women to prevent iron deficiency anemia. It may be concluded that in the studied group, a better baseline iron status and lower baseline vitamin C intake may result in a more effective dietary intervention with iron and vitamin C administered separately to improve iron status in young women.

## 1. Introduction

Anemia has been recognized by the World Health Organization (WHO) as a global health problem occurring both in developing and developed countries, which has many serious health consequences [1]. WHO estimates that anemia affects nearly two billion people worldwide [2], and it is the most frequent hematological disorder [3]. Some population groups are particularly vulnerable to its development, including infants, children under 5 years of age, and women of childbearing age [4]. Despite the multifactorial pathophysiology of anemia, the most common cause is micronutrient deficiency, with iron deficiency being the most prevalent [5]. Another possible contributor to anemia development may be gene polymorphisms [6].

Dietary iron occurs in food products in two forms, which differ in their chemical form and bioavailability: heme iron and non-heme iron [7]. Heme iron is present only in hemoglobin and myoglobin derived from animal products [8], while non-heme iron is found both in animal and plant products [9]. The bioavailability of these two forms of iron is diverse, as heme iron may be absorbed up to 30% in the human body, while the absorption of the non-heme form is influenced by other nutrients and ranges from 1% to 10% [10]. However, a predominant form of iron in an omnivorous diet is non-heme iron, which makes up 85–90% of total iron intake [11].

Women of reproductive age are at higher risk of anemia compared to men due to the increased requirement for iron, resulting from regular blood loss during menstruation [12,13], as well their specific dietary habits, namely their lower intake of meat compared to men [14]. Therefore, it is indicated that the main risk factors for anemia development in women are an improperly balanced diet and high blood loss during menstruation [15]. On this account, providing sufficient amounts of iron in everyday meals and improving the bioavailability of non-heme iron is a matter of great importance in this population group [16].

There are various studies describing the results of dietary interventions using meat in women with diagnosed anemia or low iron stores which aimed to improve their hematological parameters [17,18]. However, females display lower preference towards meat compared to males [19]. Moreover, as found in some studies, women are more conscious about the negative influence of meat on the environment [20] and therefore, they are more likely than men to reduce their meat intake [21]. It appears that plant-based dietary interventions may be a more acceptable approach for patients in improving iron status in young women, being consistent with the current dietary trends of sustainable food consumption urging reduced meat intake [22].

As ascorbic acid is a potent enhancer of non-heme iron absorption [23], some intervention studies administering products rich in vitamin C in conjunction with iron sources showed improved iron status [24,25]. This results from the fact that while iron and vitamin C are present in the small intestine simultaneously for interaction, ascorbic acid facilitates iron absorption by forming a chelate with ferric iron at acid pH that remains soluble at the alkaline pH of the duodenum [26]. However, for the time being, it is unknown whether iron and vitamin C administered separately will still be present in the small intestine simultaneously for the interaction to be effective. Taking this into account, the aim of this intervention study was to assess the effectiveness of a dietary intervention with iron and vitamin C administered separately in improving iron status in young women to prevent iron deficiency anemia.

## 2. Materials and Methods

### 2.1. Ethical Statement and Study Design

The present study was carried out at the Department of Dietetics of the Institute of Human Nutrition Sciences at Warsaw University of Life Sciences, Poland. The study was performed according to the guidelines laid down in the Declaration of Helsinki, and all procedures related to human subjects were approved by the Ethics Committee of the Faculty of Human Nutrition and Consumer Sciences of the Warsaw University of Life Sciences (no. 32/2020). All participants provided written informed consent prior to the study.

The present intervention study lasted for 8 weeks and participants received a daily supply of 50 g of iron-fortified oat flakes (as a source of iron) to be consumed with breakfast and 200 mL of orange juice (as a source of vitamin C) to be consumed separately in the second part of the day. At baseline, after 4 weeks of intervention, and after 8 weeks of intervention, parameters of iron status were controlled to assess the effectiveness of the applied intervention.

### 2.2. Studied Population

Prior to recruitment, a sample size calculation was performed. The number of women living in Warsaw or surrounding counties, according to the Polish Central Statistical Office, is 177,437 [27]. However, the population proportion was determined as 80%, given the fact than anemia prevalence in Polish women of reproductive age is approximately 20% [28]; therefore, the share of women without anemia in the general Polish population is 80% (such women were only included in the study). The confidence level was set as 90%, while the margin of error was set as 15%. Therefore, the minimum number of participants required for the present study was 19.

The study was conducted in a group of 29 women of childbearing age, aged 18–30 years. Females were recruited using a convenience sampling procedure, with the snowball effect in the period from December 2020 to January 2021. Information regarding the study was also announced via the social media of Warsaw University of Life Sciences.

The inclusion criteria were determined as follows:

− Women aged 18–30.
− Living in Warsaw or its surrounding areas (due to the necessity of regular visits to the Department of Dietetics to collect food products provided in the dietary intervention).
− Providing written informed consent to participate in the study.

The exclusion criteria were determined as follows:

− Iron deficiency anemia (diagnosed based on serum hemoglobin level <12 g/Dl, according to the WHO criteria [29]).
− Allergy or intolerance to any food products applied in the dietary intervention.
− The occurrence of diseases associated with malabsorption of nutrients, including celiac disease, inflammatory bowel diseases, and short bowel syndrome.
− The occurrence of acute and chronic bleedings, including those in the course of gastric and duodenal ulcer disease, hemophilia, esophageal varices, or pulmonary tuberculosis.
− Reaching menopause or the appearance of menopausal symptoms.
− Pregnancy or lactation.
− Applying iron supplementation.

### 2.3. Study Intervention

At the beginning of the study, participants were informed about the scope and the aim of the research and that their participation is voluntary. Study participants were provided with a daily supply of 50 g of iron-fortified oat flakes and 200 mL of orange juice to be consumed separately every day. Iron-fortified oat flakes were recommended to be consumed for breakfast and they contained 7.1 mg of iron and 18.5 mg of vitamin C per serving (50 g). Orange juice was recommended to be consumed in the second part of the day, separately from iron-fortified oat flakes, and it contained 31 mg of vitamin C per serving (200 mL). Participants were allowed to add any other habitually consumed food products to the products applied within the intervention. The intervention lasted for 8 weeks, and during it, apart from intervention products, the study group was requested to maintain regular eating habits as much as possible. Iron-fortified oat flakes were packed in 50 g portions, while orange juice was packed in 200 mL portions. Every two weeks, participants were given a supply of products for 14 days (14 × 50 g of iron-fortified oat flakes, 14 × 200 mL of orange juice).

The products used in the study were selected to represent a possible dietary intervention that could be applied to the general Polish population. Cereals and cereal products, as staple foods, are commonly consumed in Poland [30]. Moreover, as iron fortification of food is rather infrequent in Poland, primarily cereal products are fortified with iron [31], and in our previous study, we found that cereal products contribute to 28% of total daily iron intake in an average diet of young females [32]. Moreover, a portion of 50 g of oat flakes is a commonly consumed serving size among females [33]. Orange juice is a good source of vitamin C [34], which is a powerful enhancer of non-heme iron absorption [35], and simultaneously, it contains less fiber than whole orange [36], as fiber decreases iron absorption from food [37]. Additionally, the amount of orange juice consumed within this intervention was consistent with Polish recommendations [38], indicating the possibility of including one glass of fruit juice (200 mL) as one portion of fruit.

Iron-fortified oat flakes used in the study were provided by Nestlé S.A. and orange juice was provided by Tymbark–MWS by Maspex. The amount of iron and vitamin C provided in oat flakes and orange juice seemed to be effective to improve iron status, as was shown in another intervention study [39]. All products applied in the intervention in all participants were derived from the same batches. Prior to the intervention, iron-fortified oat flakes and orange juice were analyzed by one of the leading accredited food laboratories in Poland, ALAB PLUS Ltd., in terms of iron and vitamin C content. Iron content in products was determined using inductively coupled plasma mass spectrometry, and vitamin C content was determined using ultrahigh-performance liquid chromatography [40]. The mean content of iron in iron-fortified oat flakes was 14.2 ± 2.2 mg/100 g, while in orange juice, it was 0.12 ± 0.02 mg/100 mL. The average vitamin C content in iron-fortified oat flakes was 37.0 ± 4.0 mg/100 g, while in orange juice, it was 15.5 ± 2.0 mg/100 mL.

### 2.4. Measurements

#### 2.4.1. Anthropometric Measurements and Body Composition

At baseline, anthropometric measurements, including body mass and height, were conducted for every participant. Body mass was determined using a calibrated weight scale with an accuracy of ±0.1 kg, and body height was determined using a stadiometer with an accuracy of ±0.5 cm. The measurements were performed by a professional dietitian, in compliance with recommended procedures [41]. Then, the BMI was calculated using the Quetelet equation [42].

At baseline, the body composition of participants was assessed using bioelectrical impedance analysis. In order to provide reliable measurements, each participant was informed about proper preparation for the study. Women were told to avoid coffee and other caffeine beverages, avoid any alcoholic beverages, avoid any physical training the day before the measurement, and to be in a fasting state, so measurements were conducted in the morning, according to a commonly applied protocol [43]. The measurement was conducted while the participant was in a standing position, using the BC-418 MA (Tanita, Tokyo, Japan) device, according to the principles set by Kyle et al. [44]. The device measured resistance and reactance, and based on these data, fat mass, total body water, and muscle mass were calculated using GMON software version 3.2.7 (Medizin & Sevice GmbH., Chemnitz, Germany) dedicated to the analyzer.

#### 2.4.2. Iron Status

Iron status was evaluated based on red blood cells, hemoglobin, hematocrit, iron, and ferritin levels. Other hematological parameters, i.e., mean corpuscular volume (MCV), mean corpuscular hemoglobin (MCH), mean corpuscular hemoglobin concentration (MCHC), platelets, and white blood cells, were assessed with a standard complete blood count at baseline, after 4 weeks of the dietary intervention, and after 8 weeks of the dietary intervention. These parameters are commonly used in intervention studies where iron status is assessed [45,46]. At baseline, each participant had a complete blood count performed. The participant was qualified for the study if her hemoglobin level was 12.0–16.0 g/dL, interpreted as a normal value [47].

Venous blood samples were collected by a qualified nurse at a medical center in Warsaw, Poland, while participants were in a fasting state. Each blood sample was analyzed by the same person, using the same methodology, equipment, and in the same conditions. The following iron status parameters were interpreted as normal:

− Red blood cell level—3.5–5.5 × 10^12^/l [47].
− Hemoglobin level—12.0–16.0 g/dL [47].
− Hematocrit level—36–46% [47].
− Iron level—40–175 µg/dL [48].
− Ferritin level—13–150 ng/mL [49].

#### 2.4.3. Nutrients Intake

The intervention study involved daily consumption of iron-fortified oat flakes as a source of iron and orange juice as a source of vitamin C. Prior to the intervention, after 4 weeks of the intervention, and after 8 weeks of the intervention, participants’ iron and vitamin C intakes were controlled. Additionally, folate intake was controlled, as folate is the other nutrient associated with the risk of anemia [50]. Therefore, all participants were asked at that time to fill out the dedicated questionnaires assessing iron, vitamin C, and folate intakes. The data regarding iron, vitamin C, and folate intakes after 4 and 8 weeks of intervention were used to control whether, apart from the applied intervention, the intakes of the above-mentioned nutrients did not significantly change, as participants were recommended to maintain their habitual diet.

The data regarding iron intake were obtained using the previously validated IRON Intake Calculation—Food Frequency Questionnaire (IRONIC-FFQ) [51], and recalculated into iron intake using developed formulas [48] based on the information from Polish food composition tables [52]. As a result, information regarding total iron, heme-iron, non-heme iron, iron derived from animal products, and iron derived from plant products was gathered, as described in the previous study [32].

The data regarding vitamin C intake were obtained using the vitamin C-specific food frequency questionnaire comprising questions about food items rich in vitamin C (fruits, vegetables, fruit and vegetable juices, potatoes, ketchup and tomato concentrate, meat organs), included from the validated food frequency questionnaires, Ironic-FFQ [51], Iodine-FFQ [53], and Mg-FFQ [54], and recalculated into vitamin C intake using developed formulas [51,53,54] based on the information from Polish food composition tables [52].

The data regarding folate intake were obtained using the previously validated Folate-Intake Calculation—Food Frequency Questionnaire (Fol-IC-FFQ) [55], and recalculated into folate intake using developed formulas [55] based on the information from Polish food composition tables [52].

The Polish Recommended Dietary Allowance (RDA) values for women aged 18–30 were used as follows: iron, 18 mg/day; vitamin C, 75 mg/day; folate, 400 µg/day, as the study was treated as a model of intervention for individuals at risk of insufficient intake [56].

#### 2.4.4. Menstrual Blood Loss

Average menstrual blood loss during typical menstruation was evaluated based on the menstrual pictogram developed and validated by Wyatt et al. [57], being an easy-to-use, semiquantitative tool to assess typical blood loss during menstruation. Participants were given menstrual pictograms and they were informed of how to complete it to define their average menstrual blood loos. The menstrual pictogram is a tool that contains pictorial representations of graded staining from slight to severely stained tampons and sanitary napkins. Besides scoring each sanitary item, participants were also asked to determine whether it was napkin used for daytime or nighttime and whether the tampon was regular, super, or super plus, all of which are characterized by different absorption capacities. Icons representing blood lost as clots and that lost when changing tampons and napkins were also included in the menstrual pictogram. Extraneous blood loss was determined using three pictogram representations of slight, moderate, and severe blood loss while changing hygiene products. Participants were asked to note down their average blood loss each time they changed their tampon or napkin. None of the participants declared using a menstrual cup.

Each degree of staining has a different score, from 0.5 to 15.0, which corresponds to ml of lost blood during menstruation. By summing all points derived from used sanitary products, the total amount of blood lost during menstruation was calculated. The results were interpreted based on a common assumption, that normal menstrual bleeding is when blood loss per one cycle is <80 mL, while heavy menstrual bleeding is when blood loss per one cycle is ≥80 mL [58].

### 2.5. Statistical Analysis

The study participants were compared in the subsamples, stratified based on the effectiveness of the applied dietary intervention after 4 and 8 weeks, assessed for red blood cells, hemoglobin, hematocrit, iron, and ferritin levels separately.

− After 4 weeks of intervention, the intervention was classified as (1) effective if after 4 weeks (in the middle of the intervention), the level of the specific parameter of iron status was not lower than the baseline level (conducted for red blood cells, hemoglobin, hematocrit, iron, and ferritin levels separately), or (2) ineffective if after 4 weeks, the level of the specific parameter of iron status was lower than the baseline level.
− After 8 weeks of intervention, the intervention was classified as (1) effective if after 8 weeks (after intervention), the level of the specific parameter of iron status was not lower than the baseline level (conducted for red blood cells, hemoglobin, hematocrit, iron, and ferritin levels separately), or (2) ineffective if after 8 weeks, the level of the specific parameter of iron status was lower than the baseline level.

The normality of distribution was determined using a Shapiro–Wilk test. The comparison of the subsamples was performed using Student’s *t*-test or the Mann–Whitney U test (depending on distribution). For paired comparisons, we used the *t*-test for dependent samples or the Wilcoxon signed-rank test (depending on distribution).

The statistical significance was set at the level of *p* ≤ 0.05. The statistical analysis was conducted using Statistica version 13.3 (StatSoft Inc., Tulsa, OK, USA).

## 3. Results

The baseline characteristics of the participants of the study are presented in Table 1. The participants of the study were aged 21–29 years. Their mean BMI was 22.0 ± 2.5 kg/m^2^, and there were underweight individuals (*n* = 4), normal body mass individuals (*n* = 23), and overweight individuals (*n* = 2). Their median menstrual blood loss was 48.5 mL and it ranged from 16.0 mL to 112.0 mL, and there were individuals with normal bleeding (*n* = 22) and heavy bleeding (*n* = 7).

The baseline dietary intake of the participants of the study is presented in Table 2. The mean daily iron intake of the participants of the study was 11.49 ± 4.72 mg. In comparison with the RDA value, there were individuals of inadequate iron intake (*n* = 27) and adequate iron intake (*n* = 2) in the studied group. The median daily vitamin C intake of the participants of the study was 77.50 mg and it ranged from 32.86 mg to 240.71 mg. In comparison with the RDA value, there were individuals of inadequate vitamin C intake (*n* = 14) and adequate vitamin C intake (*n* = 15) in the studied group. The median of daily folate intake of the participants was 214.99 µg and it ranged from 100.28 µg to 635.73 µg. In comparison with the RDA value, there were individuals of inadequate folate intake (*n* = 25) and adequate folate intake (*n* = 4) in the studied group.

The iron status of the participants and the other hematological parameters throughout the study period are presented in Table 3. Comparing the results after 4 weeks of intervention with the results at baseline, red blood cell levels (*p* = 0.0299) and serum ferritin (*p* = 0.0128) decreased. However, comparing the results after 8 weeks of intervention with the results after 4 weeks of intervention, serum ferritin increased (*p* = 0.0189); consequently, serum ferritin results after 8 weeks of intervention did not differ from baseline (*p* > 0.05). After 8 weeks of intervention, statistically significant differences compared with baseline were only observed for hematocrit, as its level after 8 weeks of intervention was higher than for the baseline (*p* = 0.0491).

The comparison of the baseline characteristics of the subsamples (red blood cells level, dietary intake, and menstrual blood loss) in subsamples stratified by the effectiveness of dietary intervention assessed for red blood cell levels after 4 and 8 weeks is presented in Table 4. Comparing subsamples with dietary interventions considered effective and ineffective for red blood cell levels, the difference in baseline vitamin C intake was revealed, and it may be indicated that lower baseline vitamin C intake may result in a more effective dietary intervention (*p* = 0.0231).

The comparison of the baseline characteristics of the subsamples (hemoglobin level, dietary intake, and menstrual blood loss) in subsamples stratified by the effectiveness of dietary intervention assessed for hemoglobin levels after 4 and 8 weeks is presented in Table 5. Comparing subsamples with dietary interventions considered effective and ineffective for hemoglobin levels, the difference in baseline hemoglobin level was revealed, and it may be indicated that a higher hemoglobin level may result in a more effective dietary intervention (*p* = 0.0143).

The comparison of the baseline characteristics of the subsamples (hematocrit level, dietary intake, and menstrual blood loss) in subsamples stratified by the effectiveness of dietary intervention assessed for hematocrit levels after 4 and 8 weeks is presented in Table 6. Comparing subsamples with dietary interventions considered effective and ineffective for hematocrit level, the difference in baseline hematocrit levels was revealed, and it may be indicated that a higher hematocrit level may result in a more effective dietary intervention (*p* = 0.0497).

The comparison of the baseline characteristics of the subsamples (iron level, dietary intake, and menstrual blood loss) in subsamples stratified by the effectiveness of dietary intervention assessed for serum iron level after 4 and 8 weeks is presented in Table 7. Comparing subsamples with dietary interventions considered effective and ineffective for iron levels, the difference in baseline iron level was revealed, and it may be indicated that a higher serum iron level may result in a more effective dietary intervention (*p* = 0.0101).

The comparison of the baseline characteristics of the subsamples (serum ferritin level, dietary intake, and menstrual blood loss) in subsamples stratified by the effectiveness of dietary intervention assessed for serum ferritin level after 4 and 8 weeks is presented in Table 8. Comparing subsamples with dietary interventions considered effective and ineffective for serum ferritin levels, the difference in baseline serum ferritin level was revealed, and it may be indicated that a higher serum ferritin level may result in a more effective dietary intervention (*p* = 0.0343).

## 4. Discussion

Ascorbic acid effectively increases the absorption of ferrous ions (Fe^3+^) and ferric ions (Fe^2+^) [59]. It results from the reducing properties of vitamin C which make the iron soluble in a wide range of pH levels and allows iron to be absorbed through iron transporters in the small intestine [21]. According to the National Institutes of Health, the Recommended Dietary Allowance (RDA) for nonpregnant women over 19 years old is 75 mg/day [60]. However, it is indicated that increased vitamin C intake higher than the RDA has beneficial effects for long-term health outcomes, including lower risk of cardiovascular diseases [61,62]. Vitamin C intakes ranging from 100 to 200 mg/day will maintain blood concentration at adequate to saturating status (50–75 µmol/L) [63]. In low- and middle-income countries, vitamin C hypovitaminosis and deficiency is common, but in high-income settings, it is infrequent [63].

In the present study, 51.7% of study participants were characterized by adequate vitamin C intake, while 48.9% of the sample had inadequate vitamin C intake. In this study, vitamin C intake was assessed based on a dedicated Food Frequency Questionnaire (FFQ), and it should be noted that FFQs have a tendency to overestimate the intake of food products in general, and as a result, also overestimate the intake of specific nutrients [64]. Therefore, it may be supposed that the actual share of participants with inadequate vitamin C intake in the study group may have been even higher, as in the national study of Waśkiewicz et al. [65], it was indicated that 30.1% of Polish women did not consume recommended amounts of vitamin C.

However, assessing the effectiveness of dietary intervention with iron and vitamin C administered separately on red blood cells level, it was shown that the baseline vitamin C status may be a determinant of effectiveness. It was found that in women with lower baseline vitamin C intake, the dietary intervention with iron and vitamin C administered separately may be effective in improving iron status, but not in women with an adequate vitamin C status. This may stem from the fact that participants with lower baseline vitamin C intake presumably may have had vitamin C deficiency, so therefore, each intervention increasing the overall intake of vitamin C will be beneficial in improving their iron status. It is known that the potential effectiveness of vitamin C in intervention studies is associated with individuals’ vitamin C status at the beginning of the study, and people without some degree of inadequacy/deficiency of vitamin C are rather unlikely to benefit from intake or supplementation [66]. In previously conducted intervention studies in anemic women, it was revealed that the amount of vitamin C which is effective in improving iron status was 164 mg of ascorbic acid in the intervention meal [25] or 174.6 mg as a daily intake [24]. However, as the present study sample consisted of non-anemic women, it is presumed that lower doses of vitamin C would be effective in improving women’s iron status.

After 8 weeks of intervention, statistically significant differences compared to baseline were found only for hematocrit, as its level after 8 weeks of intervention was higher than the baseline. At the same time, the ferritin level after 4 weeks of intervention was significantly lower compared to baseline, but after 8 weeks of intervention, it was significantly higher than after 4 weeks of intervention. Such results indicate that the applied intervention may be effective in maintaining and even improving iron status, even if dietary intervention with iron and vitamin C administered separately is applied. Despite the fact that food products are most likely to influence iron absorption when consumed simultaneously [10], in the present study, it was shown that separate administration of products in some cases may be effective, as well.

Moreover, it was revealed that in order to effectively improve iron status, namely hematocrit and ferritin levels, long-lasting dietary interventions are needed, as increased hematocrit and ferritin levels were observed after 8 weeks of intervention, as was observed in other studies [67]. A shorter intervention period may be not only ineffective, but may also decrease iron status, as seen in the conducted study for ferritin level after 4 weeks of intervention. Serum ferritin shows the level of iron stores in the body [68]. Apart from iron deficiency, only two conditions are known to lower serum ferritin levels—hypothyroidism and vitamin C deficiency [69]. As the present study was conducted in a group of non-anemic women, one of the possible explanations for the decreased level of ferritin after 4 weeks of the dietary intervention is that the study participants may have been vitamin C-deficient, and after 8 weeks, they reached an appropriate vitamin C status, which resulted in an increase in serum ferritin level.

It may be noticed that apparently all studies that use products rich in iron and vitamin C to improve women’s iron status administered them in the same meal [24,25,70]. To our knowledge, the present study is the first where food products containing iron and vitamin C are applied separately. It may be supposed that vitamin C, even if applied separately with a product rich in iron, facilitates its mobilization from storage sites and improves iron status [71], and it was observed that it may increase the fraction of RBC with normal Hb content [72]. It also seems that in the case of women with lower intake of vitamin C, the total amount of vitamin C may be a more important factor influencing the effectiveness of applied dietary intervention than the time in which vitamin C and iron are consumed. In the typical Polish diet, fruits and vegetables are main sources of vitamin C [73,74]. In the study of Górska-Warsewicz et al. [73], it was found that vegetables and vegetable products are the main contributors to vitamin C supply (37.7% of daily supply), while in another Polish study by Rejman et al. [74], fruits and fruit products provided 23.7% of total dietary intake of vitamin C. However, it should be noted that potatoes, as one of the staple foods for the Polish population, also provide a considerable amount of vitamin C in the Polish diet (14.1% of daily supply [73]). Taking this into account, efforts should be put into increasing the overall intake of fruits and vegetables, as well as their preserves, because as shown in the studies, young Polish adults are characterized by the inadequate intake of fruits and vegetables [75].

Finally, in the conducted study, comparing subsamples with dietary interventions considered effective and ineffective for hemoglobin, hematocrit, iron, and serum ferritin levels, it was indicated that higher baseline levels of hemoglobin, hematocrit, iron, and serum ferritin may result in a more effective dietary intervention. Taking this into account, it may be indicated that a better baseline iron status within the studied group promoted a more effective dietary intervention. It should be noted that women with better baseline iron status did not have significantly higher iron intake compared to participants with worse baseline iron status. Therefore, it may be supposed that women with better baseline iron status which promoted a more effective dietary intervention may have a more efficient mechanism of iron absorption. It is indicated that there are wide intra- and inter-individual variations in iron absorption [76]. In the study of Olszon et al. [77] conducted in a homogenous group of anemic men, there were pronounced individual differences in iron absorption from food, ranging from 1.9 to 5.0 mg. Individual day-to-day differences in iron absorption also contributes to deviations [78]. However, the exact mechanisms underlying these variations in iron absorption are not fully understood, and further investigation is needed.

It should be noted that some variables may affect iron status in women of reproductive age, including heme and non-heme iron intake. In the study of Young et al. [11], it was revealed that both heme and non-heme iron were positively correlated with serum ferritin, and the heme form was found to be a stronger predictor than the non-heme form. A large cohort study by Reeves et al. [79] conducted among Australian women showed that intake of heme iron was a statistically significant predictor of iron deficiency. However, in the majority of studies that analyzed the influence of total iron intake on iron status, no association was found [80,81,82]. It appears that the type of iron (heme vs. non-heme) may be a more important determinant of iron status than total iron intake [10].

Although ascorbic acid is a powerful enhancer of non-heme iron absorption, a number of studies did not confirm any correlation between total daily vitamin C intake and iron status, which may have resulted from various interfering factors [81,83,84]. Moreover, a study of Cade et al. [85] carried out in a group of females aged 35–69 showed a negative association between fruit juice intake and iron status, but a positive association between vitamin C intake and iron status.

One of the factors that contributes to a negative iron balance in women of childbearing age is menstruation. In the study of Blanco-Rojo et al. [80], it was stated that levels of iron biomarkers, such as hemoglobin, hematocrit, ferritin, MCV, serum iron, and transferrin saturation, were significantly lower in individuals with a higher menstrual blood loss coefficient. Another study of Moschonis et al. [86] carried out on a group of pubertal girls indicated that girls with menses were 2.57 times more likely to be iron-depleted (assessed based on serum ferritin) compared to girls with no menstruation. Therefore, identifying women with high menstrual losses should be a key component of a strategy aiming at anemia and iron deficiency prevention [87].

Another important factor influencing the iron status of participants in the present study may be the place of residence (urban environment). In the study of Okafor et al. [88] conducted in pregnant women, it was found that the prevalence of iron deficiency anemia, iron depletion, and iron deficiency were significantly higher among women from rural communities compared to those from urban communities. Similar results obtained by Tesfaye et al. [89] also confirmed that iron status, namely anemia prevalence assessed based on hemoglobin level, was higher in adults living in rural areas compared to residents of urban areas.

Although the present study indicated some interesting observations that are important in terms of improving women’s iron status, its limitations must also be mentioned. First of all, the gathered sample size was relatively small, and the biological variability may have also influenced the results; therefore, they cannot be extrapolated to the general population of women. Secondly, the applied dietary intervention lasted only 8 weeks. The other issue results from the fact that within the conducted study, a placebo sub-group was not planned—a placebo-controlled trial would have allowed us to obtain more valid observations. Finally, other factors which could have possibly influenced menstrual blood loss in women such as oral contraceptive pills or intrauterine device usage were not assessed. It may be concluded that more reliable intervention studies taking into account different confounding factors, such as baseline nutritional status and menstrual patterns, are needed to effectively improve iron status in a group of women of childbearing age.

## 5. Conclusions

We concluded that dietary intervention with iron and vitamin C administered separately may be effective in improving iron status in young women to prevent iron deficiency anemia. It may be concluded that a better baseline iron status and lower baseline vitamin C intake may promote a more effective dietary intervention of iron and vitamin C administered separately in improving iron status in young women.

## Figures and Tables

**Table 1:** The baseline characteristics of the participants of the study (*n* = 29).

	Mean ± SD	Median	Min	Max
Age (years)	24.5 ± 2.0	24.0	21.0	29.0
Body weight (kg)	60.2 ± 7.1	60.3	48.2	74.7
Height (cm)	165.3 ± 5.0	165.0	156.0	175.0
BMI (kg/m^2^)	22.0 ± 2.5	22.1	18.0	29.4
Fat mass (%)	27.7 ± 5.8	27.7	15.5	40.1
Total body water (%)	52.9 ± 4.2	52.9	43.9	61.8
Muscle mass (%)	68.7 ± 5.5	68.8	57.0	80.3
Menstrual blood loss (mL)	55.1 ± 28.3	48.5 *	16.0	112.0

* nonparametric distribution (verified using Shapiro–Wilk test).

**Table 2:** Baseline dietary intake of the participants of the study (*n* = 29).

	Mean ± SD	Median	Min	Max
Iron (mg/day)	11.49 ± 4.72	10.29	3.89	20.48
Animal iron (mg/day)	2.36 ± 1.49	2.53	0.0	5.25
Plant iron (mg/day)	9.13 ± 4.97	8.78	2.47	20.48
Heme iron (mg/day)	0.94 ± 0.60	1.01	0.00	2.10
Non-heme iron (mg/day)	10.54 ± 4.77	9.33	3.32	20.48
Vitamin C (mg/day)	105.39 ± 64.42	77.50 *	32.86	240.71
Folate (µg/day)	248.90 ± 127.29	214.99 *	100.28	635.73

* nonparametric distribution (verified using Shapiro–Wilk test).

**Table 3:** The iron status of the participants of the study (*n* = 29), accompanied by the other hematological parameters, throughout the study period.

Variables—Baseline Level	t0—at Baseline		t4—after 4 Weeks of Intervention		t8—after 8 Weeks of Intervention		*p* (t0 vs. t4)	*p* (t4 vs. t8)	*p* (t0 vs. t8)
	Mean ± SD	Median (Min–Max)	Mean ± SD	Median (Min–Max)	Mean ± SD	Median (Min–Max)			
RBC	4.43 ± 0.34	4.50 (3.60–5.00)	4.50 ± 0.29	4.40 * (4.00–5.20)	4.51 ± 0.32	4.40 (4.00–5.20)	0.0299	0.8191	0.0580
Hb	13.1 ± 0.8	12.9 * (11.0–14.1)	13.3 ± 0.7	13.1 (12.1–14.4)	13.3 ± 0.8	13.3 (11.7–15.4)	0.1059	0.5532	0.0619
Ht	39.7 ± 2.4	40.0 (33.0–43.0)	40.3 ± 1.9	40.0 (37.0–44.0)	40.5 ± 2.3	40.0 (36.0–46.0)	0.0785	0.7262	0.0491
Fe	95.5 ± 45.6	95.6 (24.7–208.8)	85.3 ± 40.1	76.7 * (40.2–217.5)	93.8 ± 50.10	92.8 (25.7–215.1)	0.1919	0.3761	0.8372
SF	36.7 ± 25.8	33.3 * (7.1–119.0)	28.3 ± 22.2	20.8 * (9.1–121.0)	32.8 ± 28.6	29.5 * (7.55–156.0)	0.0128	0.0189	0.3290
MCV	89.8 ± 3.5	90.0 (81.0–95.0)	89.8 ± 4.0	90.0 (81.0–96.0)	89.8 ± 4.0	89.0 (81.0–97.0)	0.9250	1.0000	0.9447
MCH	29.6 ± 1.7	30.0 * (26.0–32.0)	29.5 ± 1.7	30.0 * (26.0–32.0)	29.6 ± 1.7	30.0 * (26.0–32.0)	0.5751	0.4446	0.8139
MCHC	32.9 ± 0.9	32.9 (30.6–34.5)	32.8 ± 0.8	32.9 (31.5–34.3)	32.9 ± 0.8	32.9 (31.4–34.3)	0.5910	0.4608	0.8068
Plt	250.6 ± 63.7	242.0 (42.0–370.0)	255.9 ± 59.7	244.0 (146.0–401.0)	255.2 ± 48.8	257.0 (146.0–344.0)	0.4919	0.9029	0.5386
WBC	6.11 ± 1.50	5.50 * (3.50–9.80)	6.59 ± 2.33	5.90 * (4.10–16.10)	6.11 ± 1.19	5.90 * (3.80–9.00)	0.2106	0.4167	0.3381

* nonparametric distribution (verified using Shapiro–Wilk test; *p* ≤ 0.05). RBC—red blood cells (mln/µL); Hb—hemoglobin (g/dL); Ht—hematocrit (%); Fe—serum iron (µg/dL); SF—serum ferritin (ng/mL); MCV—mean corpuscular volume (%); MCH—mean corpuscular hemoglobin (pg); MCHC—mean corpuscular hemoglobin concentration (g/dL); Plt—platelets (10^9^/L); WBC—white blood cells (10^9^/L).

**Table 4:** The comparison of the baseline characteristics of the subsamples (red blood cells level, dietary intake, and menstrual blood loss) in subsamples stratified by the effectiveness of dietary intervention assessed for red blood cells level after 4 and 8 weeks.

Subsamples stratified by effectiveness assessed after 4 weeks	**Variables**	**Effective Dietary Intervention (*n* = 21)**		**Ineffective Dietary Intervention (*n* = 8)**		***p***
		**Mean ± SD**	**Median (Min–Max)**	**Mean ± SD**	**Median (Min–Max)**	
	Baseline RBC	4.48 ± 0.36	4.40 (4.00–5.20)	4.58 ± 0.21	4.55 (4.30–4.90)	0.4917
	Iron (mg)	11.74 ± 4.61	10.82 (3.89–19.57)	10.19 ± 5.14	10.11 (3.89–20.48)	0.6435
	Animal iron (mg)	2.17 ± 1.49	1.80 (0.16–5.25)	2.32 ± 1.46	1.98 (0.00–5.25)	0.2769
	Plant iron (mg)	9.57 ± 4.69	9.74 (2.47–19.41)	8.21 ± 5.38	7.39 * (2.47–20.48)	0.3414
	Heme iron (mg)	0.87 ± 0.60	0.72 (0.06–2.10)	0.93 ± 0.58	0.79 (0.00–2.10)	0.2769
	Non-heme iron (mg)	10.88 ± 4.59	10.39 (3.32–19.51)	9.39 ± 5.20	9.02 (3.32–20.48)	0.5523
	Vitamin C (mg)	103.03 ± 60.12	83.93 * (32.86–240.71)	107.85 ± 75.77	74.46 * (32.86–240.71)	0.7884
	Folate (µg)	239.45 ± 130.90	214.99 * (100.28–635.73)	262.76 ± 149.90	214.99 * (100.28–635.73)	0.5419
	Menstrual blood loss (mL)	54.0 ± 29.7	44.0 (16.0–112.0)	58.1 ± 25.7	50.3 (34.0–109.0)	0.7363
Subsamples stratified by effectiveness assessed after 8 weeks	**Variables**	**Effective dietary intervention (*n* = 19)**		**Ineffective dietary intervention (*n* = 10)**		***p***
		**Mean ± SD**	**Median (min–max)**	**Mean ± SD**	**Median (min–max)**	
	Baseline RBC	4.59 ± 0.33	4.60 (4.10–5.20)	4.35 ± 0.25	4.35 (4.00–4.80)	0.0551
	Iron (mg)	10.87 ± 4.61	9.93 (3.89–19.57)	12.66 ± 4.96	13.74 (5.81–20.48)	0.3412
	Animal iron (mg)	2.10 ± 1.56	1.43 (0.16–5.25)	2.87 ± 1.28	3.07 (0.00–4.36)	0.1914
	Plant iron (mg)	8.77 ± 4.92	8.13 (2.47–19.41)	9.79 ± 5.26	10.50 (3.30–20.48)	0.6084
	Heme iron (mg)	0.84 ± 0.62	0.57 (0.06–2.10)	1.15 ± 0.51	1.23 (0.00–1.75)	0.1914
	Non-heme iron (mg)	10.03 ± 4.67	9.21 (3.32–19.51)	11.51 ± 5.04	12.44 (4.80–20.48)	0.4362
	Vitamin C (mg)	86.02 ± 55.62	66.07 * (32.86–240.71)	142.18 ± 66.69	133.75 (57.43–240.36)	0.0231
	Folate (µg)	230.94 ± 133.41	200.79 * (100.28–635.73)	283.04 ± 113.30	269.22 (139.34–503.94)	0.1484
	Menstrual blood loss (mL)	54.2 ± 29.0	44.0 (16.0–112.0)	56.8 ± 28.4	50.5 (18.0–112.0)	0.8213

* nonparametric distribution (verified using Shapiro–Wilk test; *p* ≤ 0.05). RBC—red blood cells level (mln/µL).

**Table 5:** The comparison of the baseline characteristics of the subsamples (hemoglobin level, dietary intake, and menstrual blood loss) in subsamples stratified by the effectiveness of dietary intervention assessed for hemoglobin level after 4 and 8 weeks.

Subsamples stratified by effectiveness assessed after 4 weeks	**Variables**	**Effective Dietary Intervention (*n* = 20)**		**Ineffective Dietary Intervention (*n* = 9)**		***p***
		**Mean ± SD**	**Median (Min–Max)**	**Mean ± SD**	**Median (Min–Max)**	
	Baseline Hb	13.33 ± 0.77	13.30 (11.70–15.20)	13.30 ± 0.96	13.10 (12.20–15.40)	0.9410
	Iron (mg)	12.07 ± 4.48	12.01 (3.89–19.57)	10.19 ± 5.27	7.83 (5.24–20.48)	0.3314
	Animal iron (mg)	2.21 ± 1.52	2.17 (0.16–5.25)	2.70 ± 1.45	2.83 (0.00–4.54)	0.4242
	Plant iron (mg)	9.86 ± 4.62	10.11 (2.47–19.41)	7.49 ± 5.61	4.93 * (3.30–20.48)	0.1643
	Heme iron (mg)	0.88 ± 0.61	0.87 (0.06–2.10)	1.08 ± 0.58	1.13 (0.00–1.82)	0.4242
	Non-heme iron (mg)	11.19 ± 4.47	11.25 (3.32–19.51)	9.11 ± 5.36	6.29 * (4.67–20.48)	0.1949
	Vitamin C (mg)	106.54 ± 59.44	86.79 * (35.00–240.71)	102.84 ± 78.24	71.43 * (32.86–240.36)	0.7237
	Folate (µg)	246.41 ± 130.25	222.94 * (106.09–635.73)	254.45 ± 127.92	200.79 (100.28–503.94)	0.9812
	Menstrual blood loss (mL)	55.2 ± 30.0	46.5 (16.0–112.0)	54.9 ± 25.8	48.5 (30.0–109.0)	0.9825
Subsamples stratified by effectiveness assessed after 8 weeks	**Variables**	**Effective dietary intervention (*n* = 20)**		**Ineffective dietary intervention (*n* = 9)**		***p***
		**Mean ± SD**	**Median (min–max)**	**Mean ± SD**	**Median (min–max)**	
	Baseline Hb	13.56 ± 0.77	13.45 (12.50–15.40)	12.78 ± 0.69	12.70 (11.70–13.60)	0.0143
	Iron (mg)	11.38 ± 4.43	10.55 (3.89–19.57)	11.72 ± 5.60	9.50 (5.81–20.48)	0.8595
	Animal iron (mg)	2.17 ± 1.56	1.61 (0.16–5.25)	2.78 ± 1.31	2.87 (0.00–4.36)	0.3220
	Plant iron (mg)	9.21 ± 4.66	9.26 (2.47–19.41)	8.95 ± 5.91	6.63 (3.30–20.48)	0.8993
	Heme iron (mg)	0.87 ± 0.63	0.64 (0.06–2.10)	1.11 ± 0.52	1.15 (0.00–1.75)	0.3220
	Non-heme iron (mg)	10.51 ± 4.46	9.86 (3.32–19.51)	10.61 ± 5.69	8.36 (4.80–20.48)	0.9582
	Vitamin C (mg)	97.91 ± 63.65	69.46 * (32.86–240.71)	122.01 ± 66.72	103.93 (57.14–240.36)	0.3108
	Folate (µg)	250.75 ± 144.14	211.44 * (100.28–635.73)	244.81 ± 85.65	230.89 (139.34–412.57)	0.6886
	Menstrual blood loss (mL)	55.5 ± 29.0	44.5 (16.0–112.0)	54.3 ± 28.4	49.0 (18.0–109.0)	0.9166

* nonparametric distribution (verified using Shapiro–Wilk test; *p* ≤ 0.05). Hb—hemoglobin level (g/dL).

**Table 6:** The comparison of the baseline characteristics of the subsamples (hematocrit level, dietary intake, and menstrual blood loss) in subsamples stratified by the effectiveness of dietary intervention assessed for hematocrit level after 4 and 8 weeks.

Subsamples stratified by effectiveness assessed after 4 weeks	**Variables**	**Effective Dietary Intervention (*n* = 23)**		**Ineffective Dietary Intervention (*n* = 6)**		***p***
		**Mean ± SD**	**Median (Min–Max)**	**Mean ± SD**	**Median (Min–Max)**	
	Baseline Ht	40.4 ± 2.4	40.0 (36.0–46.0)	40.7 ± 1.8	40.5 (38.0–43.0)	0.7973
	Iron (mg)	12.05 ± 4.89	10.82 (3.89–20.48)	9.33 ± 3.56	8.44 (5.81–13.70)	0.2158
	Animal iron (mg)	2.23 ± 1.61	1.80 (0.00–5.25)	2.87 ± 0.79	2.77 (1.66–3.84)	0.3621
	Plant iron (mg)	9.82 ± 5.18	9.74 (2.47–20.48)	6.47 ± 3.11	5.79 (3.30–10.49)	0.1441
	Heme iron (mg)	0.89 ± 0.65	0.72 (0.00–2.10)	1.15 ± 0.32	1.11 (0.66–1.54)	0.3621
	Non-heme iron (mg)	11.16 ± 4.94	10.39 (3.32–20.48)	8.19 ± 3.37	7.38 (4.80–12.16)	0.1786
	Vitamin C (mg)	101.68 ± 57.66	83.93 * (32.86–240.36)	119.61 ± 91.10	74.29 * (40.00–240.71)	0.8505
	Folate (µg)	236.25 ± 122.17	214.99 * (100.28–635.73)	297.40 ± 146.64	234.31 (167.96–503-94)	0.4040
	Menstrual blood loss (mL)	53.6 ± 29.1	44.0 * (16.0–112.0)	61.1 ± 26.4	50.3 (34.0–102.0)	0.4510
Subsamples stratified by effectiveness assessed after 8 weeks	**Variables**	**Effective dietary intervention (*n* = 20)**		**Ineffective dietary intervention (*n* = 9)**		***p***
		**Mean ± SD**	**Median (min–max)**	**Mean ± SD**	**Median (min–max)**	
	Baseline Ht	41.0 ± 2.3	40.5 (36.0–46.0)	39.2 ± 1.7	40.0 (36.0–42.0)	0.0497
	Iron (mg)	11.79 ± 4.81	10.55 (3.89–20.48)	10.81 ± 4.74	7.97 * (5.81–17.18)	0.5877
	Animal iron (mg)	2.18 ± 1.58	1.61 (0.00–5.25)	2.78 ± 1.26	2.83 (0.20–4.36)	0.3259
	Plant iron (mg)	9.62 ± 5.31	9.26 (2.47–20.48)	8.04 ± 4.18	7.77 (3.30–12.90)	0.4380
	Heme iron (mg)	0.87 ± 0.63	0.64 (0.00–2.10)	1.11 ± 0.50	1.13 (0.08–1.75)	0.3259
	Non-heme iron (mg)	10.92 ± 4.96	9.86 (3.32–20.48)	9.70 ± 4.48	7.89 (4.80–15.44)	0.5333
	Vitamin C (mg)	100.78 ± 71.09	66.79 * (32.86–240.71)	115.63 ± 48.49	103.93 (57.14–188.93)	0.2116
	Folate (µg)	256.40 ± 146.71	211.44 * (100.28–635.73)	232.26 ± 71.67	230.89 (115.01–327.01)	0.8320
	Menstrual blood loss (mL)	57.6 ± 31.5	44.5 (16.0–112.0)	53.0 ± 27.5	48.8 (16.0–112.0)	0.8320

* nonparametric distribution (verified using Shapiro–Wilk test; *p* ≤ 0.05). Ht—hematocrit level (%).

**Table 7:** The comparison of the baseline characteristics of the subsamples (iron level, dietary intake, and menstrual blood loss) in subsamples stratified by the effectiveness of dietary intervention assessed for serum iron level after 4 and 8 weeks.

Subsamples stratified by effectiveness assessed after 4 weeks	**Variables**	**Effective Dietary Intervention (*n* = 11)**		**Ineffective Dietary Intervention (*n* = 18)**		***p***
		**Mean ± SD**	**Median (Min–Max)**	**Mean ± SD**	**Median (Min–Max)**	
	Baseline Fe	76.3 ± 51.6	61.1 (31.3–215.1)	104.4 ± 47.4	112.0 (25.7–181.9)	0.0835
	Iron (mg)	10.86 ± 4.63	10.29 (3.89–19.57)	11.87 ± 4.87	11.70 (5.10–20.48)	0.5876
	Animal iron (mg)	2.24 ± 1.02	2.53 (0.16–3.64)	2.44 ± 1.74	2.27 (0.00–5.25)	0.7308
	Plant iron (mg)	8.63 ± 4.94	8.13 (2.47–19.41)	9.43 ± 5.10	9.32 (3.22–20.48)	0.6806
	Heme iron (mg)	0.89 ± 0.41	1.01 (0.06–1.46)	0.98 ± 0.70	0.91 (0.00–2.10)	0.7308
	Non-heme iron (mg)	9.97 ± 4.73	9.21 (3.32–19.51)	10.89 ± 4.89	10.75 (4.64–20.48)	0.6213
	Vitamin C (mg)	104.49 ± 67.03	77.50 (35.89–240.71)	105.94 ± 64.73	77.68 * (32.86–240.36)	0.8397
	Folate (µg)	253.01 ± 161.31	194.89 * (109.53–635.73)	246.39 ± 106.59	254.45 (100.28–503.94)	0.6052
	Menstrual blood loss (mL)	67.4 ± 31.3	63.0 * (18.0–112.0)	47.6 ± 24.2	38.5 (16.0–66.0)	0.0919
Subsamples stratified by effectiveness assessed after 8 weeks	**Variables**	**Effective dietary intervention (*n* = 15)**		**Ineffective dietary intervention (*n* = 14)**		***p***
		**Mean ± SD**	**Median (min–max)**	**Mean ± SD**	**Median (min–max)**	
	Baseline Fe	116.1 ± 51.0	111.0 (37.5–215.1)	69.8 ± 37.6	61.8 (25.7–133.2)	0.0101
	Iron (mg)	12.72 ± 5.47	13.78 (3.89–20.48)	10.16 ± 3.49	9.67 (5.24–16.03)	0.1484
	Animal iron (mg)	2.34 ± 1.53	2.72 (0.00–4.36)	2.39 ± 1.51	2.15 (0.20–5.25)	0.9238
	Plant iron (mg)	10.39 ± 6.12	11.14 (2.47–20.48)	7.77 ± 3.00	7.95 (3.30–13.17)	0.1609
	Heme iron (mg)	0.93 ± 0.61	1.09 (0.00–1.75)	0.96 ± 0.61	0.86 (0.08–2.10)	0.9238
	Non-heme iron (mg)	11.79 ± 5.69	12.72 (3.32–20.48)	9.21 ± 3.22	8.91 (4.67–13.93)	0.1486
	Vitamin C (mg)	114.63 ± 72.92	83.93 * (35.00–240.71)	95.49 ± 54.83	74.46 * (32.86–231.25)	0.8786
	Folate (µg)	279.94 ± 138.18	244.99 (106.09–635.73)	215.66 ± 109.70	180.15 * (100.28–503.94)	0.1112
	Menstrual blood loss (mL)	55.6 ± 32.3	48.5 (16.0–109.0)	54.6 ± 24.4	48.5 (26.0–112.0)	0.9318

* nonparametric distribution (verified using Shapiro–Wilk test; *p* ≤ 0.05). Fe—iron level (µg/dL).

**Table 8:** The comparison of the baseline characteristics of the subsamples (serum ferritin level, dietary intake, and menstrual blood loss) in subsamples stratified by the effectiveness of dietary intervention assessed for serum ferritin level after 4 and 8 weeks.

Subsamples stratified by effectiveness assessed after 4 weeks	**Variables**	**Effective Dietary Intervention (*n* = 7)**		**Ineffective Dietary Intervention (*n* = 22)**		***p***
		**Mean ± SD**	**Median (Min–Max)**	**Mean ± SD**	**Median (Min–Max)**	
	Baseline SF	51.4 ± 50.7	23.5 (12.5–156.0)	26.9 ± 14.3	29.8 (7.6–50.0)	0.3081
	Iron (mg)	11.65 ± 4.04	13.21 (5.10–17.18)	11.44 ± 5.01	9.81 (3.89–20.48)	0.9192
	Animal iron (mg)	3.21 ± 1.13	3.64 (1.14–4.36)	2.09 ± 1.51	1.73 (0.00–5.25)	0.0826
	Plant iron (mg)	8.44 ± 3.52	9.86 (3.96–12.82)	9.35 ± 5.40	8.46 (2.47–20.48)	0.6809
	Heme iron (mg)	1.29 ± 0.45	1.46 (0.46–1.75)	0.84 ± 0.61	0.69 (0.00–2.10)	0.0826
	Non-heme iron (mg)	10.36 ± 3.80	12.12 (4.64–15.44)	10.60 ± 5.12	9.27 (3.32–20.48)	0.9118
	Vitamin C (mg)	140.08 ± 83.87	149.64 (40.00–240.71)	94.35 ± 54.72	74.46 (32.86–240.36) *	0.2961
	Folate (µg)	317.37 ± 125.27	323.90 (186.47–503.94)	227.12 ± 122.72	197.84 (100.28–635.73) *	0.0560
	Menstrual blood loss (mL)	59.9 ± 30.7	49.0 (16.0–102.0)	53.6 ± 28.1	46.3 (18.0–112.0)*	0.5751
Subsamples stratified by effectiveness assessed after 8 weeks	**Variables**	**Effective dietary intervention (*n* = 14)**		**Ineffective dietary intervention (*n* = 15)**		***p***
		**Mean ± SD**	**Median (min–max)**	**Mean ± SD**	**Median (min–max)**	
	Baseline SF	43.4 ± 36.2	35.4 (12.5–156.0)	23.0 ± 14.3	17.5 (7.6–50.0)	0.0343
	Iron (mg)	11.61 ± 4.02	12.01 (5.10–17.75)	11.37 ± 5.44	9.70 (3.89–20.48)	0.8938
	Animal iron (mg)	2.80 ± 1.35	2.85 (0.65–4.54)	1.95 ± 1.54	1.66 (0.00–5.25)	0.1275
	Plant iron (mg)	8.81 ± 4.22	9.80 (3.22–17.10)	9.42 ± 5.71	8.13 (2.47–20.48)	0.7478
	Heme iron (mg)	1.12 ± 0.54	1.14 (0.26–1.82)	0.78 ± 0.62	0.66 (0.00–2.10)	0.1275
	Non-heme iron (mg)	10.49 ± 4.05	11.25 (4.64–17.49)	10.59 ± 5.50	9.21 (3.32–20.48)	0.9565
	Vitamin C (mg)	104.55 ± 70.77	66.43 * (35.00–240.71)	106.17 ± 60.39	89.64 (32.86–240.36)	0.5557
	Folate (µg)	265.97 ± 113.43	239.45 (106.09–503.94)	232.97 ± 141.04	186.30 * (100.28–635.73)	0.2301
	Menstrual blood loss (mL)	57.3 ± 30.8	46.8 (16.0–112.0)	53.1 ± 26.7	52.0 (18.0–109.0)	0.7027

* nonparametric distribution (verified using Shapiro–Wilk test; *p* ≤ 0.05). SF—serum ferritin level (ng/mL).
